# Simultaneous Extraction and Determination of Characteristic Steroidal Saponins and Homoisoflavonoids in Zhejiang *Ophiopogon japonicus*

**DOI:** 10.3390/molecules27217380

**Published:** 2022-10-30

**Authors:** Yaoyao Zhu, Liling Wang, Meixu Chen, Yifeng Zhou, Jun Huang

**Affiliations:** 1 School of Biological and Chemical Engineering, Zhejiang University of Science and Technology, Hangzhou 310023, China; 2 Zhejiang Academy of Forestry, Hangzhou 310023, China; 3 Zhejiang Provincial Key Lab for Chemical and Biological Processing Technology of Farm Produces, Hangzhou 310023, China; 4 Zhejiang Province Collaborative Innovation Center of Agricultural Biological Resources Biochemical Manufacturing, Hangzhou 310023, China

**Keywords:** ionic liquid (IL), Zhejiang *Ophiopogon* *japonicus* (ZOJ), steroidal saponins, homoisoflavonoids, HPLC-DAD-ELSD

## Abstract

Zhejiang *Ophiopogon* *japonicus* (ZOJ) is a specific variety of *Ophiopogon japonicus* with characteristic steroidal saponins and homoisoflavonoids, which are also main pharmacodynamic constituents with clinical effects, including curing inflammation and cardiovascular diseases. However, few analysis methods were applied to simultaneously and quantitatively determine two kinds of its constituents, and hazardous organic solvents are mostly used for extraction. In this study, a new validated simultaneous extraction and determination method for four characteristic steroidal saponins and homoisoflavonoids in ZOJ was established by ionic liquid–ultrasonic extraction (IL-UAE) combined with HPLC-DAD-ELSD analysis, which can be used for the quality control of ZOJ. Chromatographic separation was performed with a DAD wavelength at 296 nm, and the ELSD parameters of the drift tube temperature (DTT), atomizer temperature (AT), and nitrogen gas pressure (NGP) were set at 20% heating power, 70 °C, and 25 psi, respectively. The optimal IL-UAE conditions were 1 mol/L [Bmim]CF_3_SO_3_ aqueous solution, a liquid–material ratio of 40 mL/g, and an ultrasonic time of 60 min. The proposed method is reliable, reproducible, and accurate, which were verified with real sample assays. Consequently, this work will be helpful for the quality control of ZOJ. It can also present a promising reference for the simultaneous extraction and determination of different kinds of constituents in other medicinal plants.

## 1. Introduction

The tuber root of *Ophiopogon japonicus* (Thunb.) Ker-Gawl (Liliaceae) is one of the most widely used traditional Chinese medicines (TCM) to cure acute and chronic inflammation and cardiovascular diseases [1,2,3]. *O. japonicus* is widely distributed in China, and *O. japonicus* cultivated in Sichuan (COJ) and Zhejiang provinces (ZOJ) are currently the two major producing varieties [4,5]. Previous studies showed that steroidal saponins and homoisoflavonoids were the two main constituents in both ZOJ and COJ; however, they had different bioactive constituents. In ZOJ, Cixi-ophiopogon B (S1) and Cixi-ophiopogon C (S2) are two unique saponins, while methylophiopogonanone A (H1) and methylophiopogonanone B (H2) are the two flavonoids with the highest content. They are crucial in the quality control of ZOJ and make ZOJ different from COJ in pharmacological activities. They can be considered as a key factor in efficacy studies and further clinical application of different varieties of *O. japonicus* [4,5,6,7,8,9]. ZOJ has long been considered as one of the characteristic and representative herbal medicines in Zhejiang province and is also recognized as the better variety among *O. japonicus* [10]. However, the cultivation of ZOJ has drastically decreased in the last 20 years, accounting for less than 5% of total output [4]. Therefore, a quantitative and qualitative analyses of the active and special constituents are of great significance for the variety protection of ZOJ.

Before quantitative analysis, sample extraction and enrichment should be performed. The selection of the extraction solvent is a key factor due to the hydrophilicity of steroidal saponins and lipophilicity of homoisoflavonoids. Methanol was a common extraction solvent in the previous determination method; however, it is strongly discouraged in the extraction process owing to its high toxicity, strong volatility, and flammability. Recently, ionic liquid (IL) as a novel and eco-friendly type of solvent with good thermal stability and high solubility to different compounds has been exploited in extraction pretreatment for HPLC determination [11,12,13,14]. Additionally, an IL aqueous solution may be a good choice for two kinds of active constituent extraction [15,16,17]. Previously, HPLC-DAD [18,19,20,21], HPLC-ELSD [22,23,24], or HPLC-MS [25,26,27] have been established for the quantitative determination of some characteristic steroidal saponins or homoisoflavonoids in ZOJ or COJ. However, steroidal saponins cannot be detected by HPLC-DAD, and homoisoflavonoids can be determined by HPLC-ELSD, albeit with relatively lower sensitivity. Recently, HPLC-UV-ELSD [8,28] and HPLC-MS [4,6,7,29] have been applied to fingerprint analyses of steroidal saponins and homoisoflavonoids in ZOJ or COJ. However, few studies on the simultaneous quantitative determination of the two kinds of characteristic compounds of ZOJ have been reported.

In this study, a new IL ultrasonic extraction (IL-UAE) process combined with the HPLC-DAD-ELSD method was developed to analyze four characteristic steroidal saponins and homoisoflavonoids in ZOJ roots. HPLC-DAD-ELSD chromatographic conditions, particularly the ELSD parameters, were optimized first. Then, single-factor experiments were used to investigate the effects of IL ultrasonic extraction parameters. Finally, the proposed method was validated and applied to the samples of ZOJ roots.

## 2. Results and Discussion

### 2.1. Optimization of HPLC Conditions

A chromatographic column with sub-3 μm core-shell particles has been developed, which offers higher speeds with minor increases in operating pressures [30]; therefore, it can be applied to normal HPLC devices. Compared with normal chromatographic columns that are 250 mm in length and 5 µm in particle sizes, the chromatograms of the Welch BoltimateTM-C_18_ column (4.6 mm × 100 mm, 2.7 µm) showed a higher resolution and shorter separation time. Therefore, we selected this for our study.

**Figure 1:**
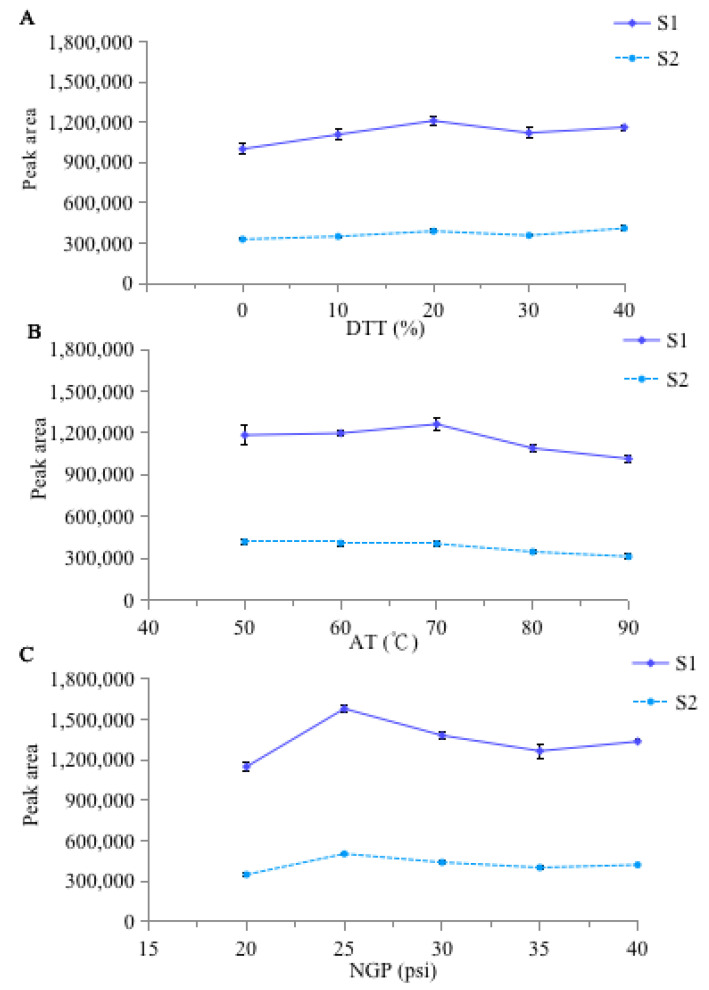
Effects of DTT (**A**), AT (**B**), and NGP (**C**) on ELSD signal response.

In the diode array detector (DAD), the detection wavelength was selected as 296 nm according to the basis of the maximum absorption wavelength (λ_max_) of two homoisoflavonoids in the UV spectra, which was consistent with previous research on the two compounds [8]. In the evaporative light-scattering detector (ELSD), the influencing factors of the drift tube temperature (DTT; expressed as ranging from 0% to 100% heating power), the atomizer temperature (AT), and the nitrogen gas pressure (NGP) were investigated and optimized by single-factor experiments. As shown in Figure 1A,B, the response signal of the peak areas initially enhanced with DTT and AT increased; however, the signal values decreased when the DTT and AT were higher than 70 °C and 20%, respectively. Similarly, when the NGP increased higher than 25 psi, the peak area significantly decreased (Figure 1C). These results were consistent with the effects of the three parameters on ELSD described in previous research [31,32]. Considering the response and noise, the optimal parameters of DTT, AT, and NGP were set at 20%, 70 °C, and 25 psi, respectively. In addition, a sustainable way of changing gain was applied to avoid the interference of polar impurities and enhance the response signal values of two steroidal saponins in ELSD: the gain was set as 1 from 0~5 min, and then it was changed to 500 from 5~25 min.

### 2.2. Optimization of IL Ultrasonic Extraction Conditions

#### 2.2.1. Type of IL

**Figure 2:**
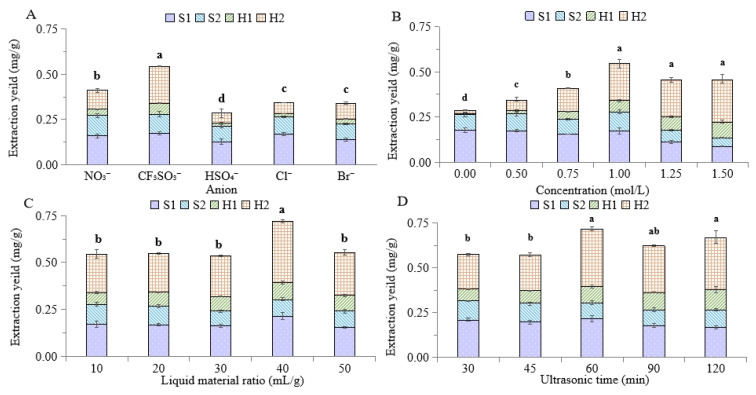
Effect of IL anion (**A**), IL concentration (**B**), liquid-material ratio (**C**), and ultrasonic time (**D**) on extraction yield. Different lowercase letters (a, b, c, d) indicated significant difference in extraction yield between different single factor level (*p* < 0.05).

It was reported that the performance of IL extraction could be greatly affected by the structures of cations and anions [33,34]. Additionally, the mechanism of extraction is very different from those of traditional organic solvents. There may be multiple forces between IL and the target extraction compounds, including hydrogen bonding, electrostatic force, π-π interaction, etc., which can strengthen the binding between ionic liquids and compounds, promote the mass transfer process, and even improve the selectivity of the extraction process [35]. Therefore, it is of paramount importance to find a suitable IL. As reported in previous studies [35,36], the lipophilicity of IL will continue to increase with the length of the alkyl chain of cation. When the length of the cationic alkyl chain is greater than five carbons, the spatial conflict becomes a greater influence than the increase in lipophilicity, resulting in a decrease in the extraction yield. So, the same cation of [Bmim]^+^ with a four-carbon-alkyl chain was selected for IL-UAE. Comparatively, the anion of IL had a more obvious impact on extraction efficiency because the degree of interaction between water and ILs is strongly dependent on anionic properties [37,38]. The effects of five different anions on the extraction efficiency for four constituents were investigated in Figure 2A. With the same cation, the anion of CF_3_SO_3_^−^ showed the highest extraction yield for two homoflavonoids, which was more than two times that of other ILs. As for the two steroidal saponins, different anions exhibited varying extraction performances; furthermore, [Bmim]NO_3_^−^ and [Bmim]HSO_4_^−^ showed the maximum and minimum extraction yields, respectively. Thus, considering the best total extraction yield for four constituents, [Bmim]CF_3_SO_3_ was finally determined as IL for the subsequent single-factor experiment.

#### 2.2.2. IL Concentration

As shown in Figure 2B, water with 0% IL concentration is not a good solvent for extracting homoflavonoids. Still, it suggested that it had an important effect for saponins extraction, which may be attributed to the principle of the dissolution in the similar material structure. With the increase in IL concentration, the extraction yields of two homoflavoniods obviously increased from 0 to 1 mol/L, and then the change became not significant from 1 to 1.5 mol/L. On the contrary, with the increase in IL from 0 to 1 mol/L, the extraction yields of two saponins did not significantly vary; however, they significantly decreased with higher IL concentrations. These results suggested IL concentration to be a key factor for ZOJ homoflavoniods extraction; however, the increase in IL concentration will increase the viscosity of the extract system [39,40], which is not conducive to the dissolution of saponins and consequently leads to the decrease in extraction yield for the two saponins [41]. Considering the total extraction yield of the four compounds from ZOJ, 1 mol/L [Bmim]CF_3_SO_3_ aqueous solution was selected as a suitable IL concentration for UAE extraction.

#### 2.2.3. Liquid–Material Ratio

In IL-UAE, the liquid–material ratio is also an important factor in achieving a high extraction yield. In this study, the extraction effect was investigated with different liquid–solid ratios of 10, 20, 25, 40, and 50 mL/g. Figure 2C indicated the change of extraction performance for four target constituents was not obvious as the IL type and concentration, which also suggested the good effect of the previous optimization design. However, the total extraction yield increased with the increase in the liquid–solid ratio from 10 to 40 mL/g, and then decreased with the further increase in the liquid–solid ratio to 50 mL/g. This trend was consistent with some previous studies [42]. The reason was that the initial increase in the liquid–solid ratio would promote the probability of contact between the target molecule and the solvent [43], but excessive liquid–solid ratio would lead to the dissolution of impurities [44]. Therefore, the liquid–material ratio was determined to be 40 mL/g.

#### 2.2.4. Ultrasonic Time

Five different levels of ultrasonic time from 30 to 120 min were tested to obtain the optimal extraction efficiency for the target compounds (Figure 2D). The extension of the ultrasound time had a positive effect on the extraction of the two flavonoids; however, its effect on saponins was relatively insignificant. Moreover, with the further extension of time after 60 min, the extraction yields of two saponins slightly decreased, which is similar to the results of previous research [40,45]; therefore, the total yield of the four compounds did not significantly change (*p* > 0.05). Considering the UAE time and energy saving, 60 min was chosen as the optimal ultrasound time. Overall, the optimum IL-UAE conditions for the four characteristic constituents from ZOJ were as follows: 1 mol/L [Bmim]CF_3_SO_3_ aqueous solution with a solid–liquid ratio of 40 mL/g and ultrasonic time of 60 min.

#### 2.2.5. Comparison of Extraction Solvent

**Figure 3:**
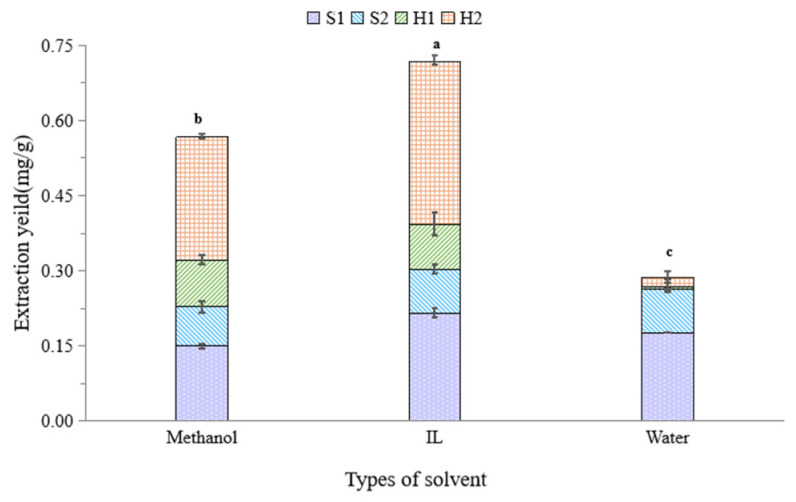
Comparison of extraction solvents on extraction yield. Different lowercase letters (a, b, c) indicated significant difference in extraction yield between different types of solvent with the same ZOJ tuber sample (*p* < 0.05).

Due to its extensive dissolution, methanol is the most common solvent used for TCM extraction for HPLC determination. With the above optimal UAE conditions except for the solvent, the IL extraction showed a relatively higher total extraction yield for the four characteristic constituents (Figure 3), which may be mainly attributed to the better extraction yield for S1 and H2. In addition, when using IL aqueous solution as an extraction solvent, there is no need for the SPE preparation step of solvent evaporation and redissolution in water [8]. Therefore, the proposed IL-UAE method is a green and effective approach to the extraction of characteristic constituents in ZOJ.

### 2.3. Method Validation

#### 2.3.1. Linearity, LOD, and LOQ

The linearity parameter is one of the most important criteria of the validation study [46]. The parameters should be determined according to the concentration or content ranges in the real sample. In our study, for the two steroidal saponins S1 and S2, their linear regression was calculated by the logarithm values of peak area (logY) and the concentration of the target analyte (logX). For the two homoisoflavonoids H1 and H2, their linear regression was fitted based on the values of the peak area (Y) and concentration of the target analyte (X). The regression equation, correlation coefficient (R^2^), and linear range along with the LODs and LOQs for the four constituents are shown in Table 1. The results indicated that each constituent showed good linearity with R^2^ greater than 0.99 within the test range. The LODs and LOQs of homoisoflavonoids are significantly smaller than those of saponins, which also reflected the better determination sensitivity of the DAD compared to ELSD [47].

#### 2.3.2. Precision, Repeatability, and Stability

The intra- precision and inter-day precision rates for the target analytes were 2.13–4.63% and 1.27–4.71% (RSD), respectively. These data suggested that the analysis of the HPLC-DAD-ELSD method had high precision in the linearity ranges. Additionally, the repeatability of the four constituents was 1.87–4.97% (RSD), which proved that this IL-UAE-HPLC method was reproducible. Moreover, the stability ranges between 2.85 and 3.73% (RSD) indicated that the four analytes in the real sample were stable for at least 3 days when stored at 4°C away from light. All of these experimental results are shown in Table 2.

#### 2.3.3. Accuracy

Table S1 reported the average recoveries of the target analytes when spiking three different known concentration levels of mixed standards. The average recoveries were between 83.88% and 106.76%, and all RSDs were less than 5%. Thus, the current method was reliable with acceptable accuracy.

#### 2.3.4. Analysis of Real Samples

**Figure 4:**
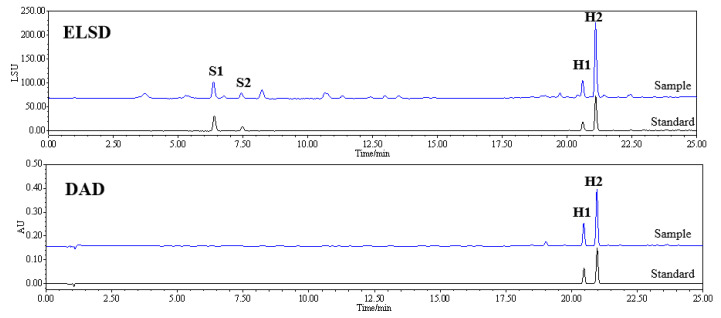
HPLC-DAD-ELSD chromatograms: mixed standard solution and typical ZOJ tuber sample on ELSD and DAD (296 nm): (S1) Cixi-ophiopogon B, (S2) Cixi-ophiopogon C, (H1) Methylophiopogonanone A, and (H2) Methylophiopogonanone B.

In our study, six other ZOJ tuber and fibrous root samples from three different planting areas were analyzed to evaluate our established method. The typical chromatograms of the standard and real sample are shown in Figure 4. It can be seen in Table 3 that four characteristic constituents were detected in all samples, and their contents were quite different from each other. Previous studies have indicated that the constituents of the fibrous roots of ZOJ were similar to those in the tuber roots of ZOJ [48,49]. Our analysis results further indicated that the contents of the four characteristic steroidal saponins and homoisoflavonoids in fibrous roots were obviously higher than those in tuber roots. Therefore, the fibrous root of ZOJ can be an important resource for research and development in functional food and pharmaceutical applications.

## 3. Materials and Methods

### 3.1. Materials

All samples, including dried tuber roots and fibrous roots of ZOJ, were collected from different plantation farms in Cixi City in the province of Zhejiang, China. Before analysis, the samples should be washed and dried to a constant weight at 50 °C. Then, these samples were grinded into powders and gathered after passing through a 40-mesh sieve. All of these powders were kept in the desiccator until analysis.

All the ionic liquids used in this study, including 1-butyl-3-methylimidazole nitrate ([Bmim]NO_3_), 1-butyl-3-methylimidazole hydrogen sulfate ([Bmim]HSO_4_), 1-butyl-3-methylimidazole trifluoromethanesulfonate ([Bmim]CF_3_SO_3_), 1-butyl-3-methylimidazole bromide ([Bmim]Br), and 1-butyl-3-methylimidazole chloride ([Bmim]Cl) were purchased from the Aladdin reagent company. HPLC-grade acetonitrile was purchased from the TEDIA Company, Inc. (Fairfield, OH, USA). The HPLC water was obtained from Wahaha Co., Ltd. The other solvents utilized for the preparation of the samples of ZOJ were all of analytical grade and were purchased from Lingfeng Chemical Reagent Co. Ltd., Shanghai, China.

**Figure 5:**
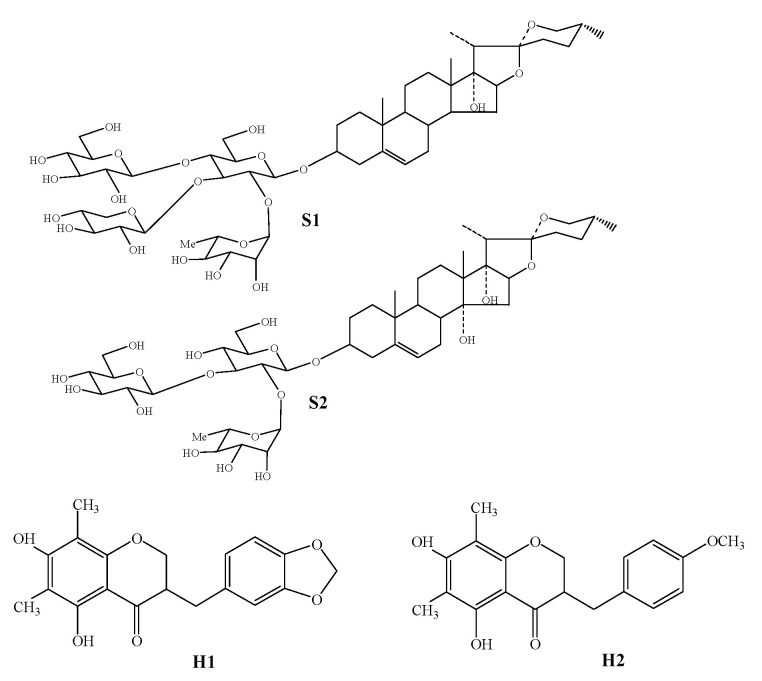
Chemical structures of four characteristic constituents in ZOJ: Cixi-ophiopogon B (S1), Cixi-ophiopogon C (S2), Methylophiopogonanone A (H1), and Methylophiopogonanone B (H2).

The characteristic ZOJ standards, including S1, S2, H1, and H2, were prepared in our laboratory [48,49], and their chemical structures are illustrated in Figure 5. The purity of these standards was >98%, which was further confirmed by HPLC.

### 3.2. Chromatographic Conditions

HPLC-DAD-ELSD analysis was performed on a Waters Alliance e2695 separation system (Waters Co., Milford, MA, USA), including a quaternary pump, an autosampler, a column oven, and a Waters 2998 diode array detector coupled with a Waters 2424 evaporative light-scattering detector. In this study, a Welch Boltimate^TM^-C_18_ column (4.6 mm × 100 mm, 2.7 µm) was selected after our preliminary tests. The column temperature was maintained at 35 °C, and the mobile phase flow rate was 1 mL/min. The mobile phase consisted of acetonitrile (A) and 0.1% formic acid (B). The gradient elution procedure was as follows: 30–40% A at 0–15 min, 40–80% A at 15–25 min, and column washing and re-equilibration for 8 min. The injection volume was 20 μL.

### 3.3. Sample Preparation

IL extraction was performed on the KQ-300DE ultrasonic apparatus (Kunshan Ultrasonic Instruments Manufacture Ltd., Jiangsu, China). About 0.25 g of sample powder and 10 mL of [Bmim]CF_3_SO_3_ with a concentration of 1 mol/L were added into a 10 mL sample flask with cover. In order to obtain more stable extraction conditions, the ultrasonic apparatus was pre-heated to 30 °C. Then, the ultrasonic extraction was performed for 60 min at 300 W.

After IL ultrasonic extraction, all extract samples are performed as follows: Firstly, the extract was centrifuged, and the supernatant (5 mL) was subjected to a solid phase extraction (SPE) column (Waters, Sep-Pak C18 3cc Vac Cartridge, 500 mg) sequentially eluting with water (5 mL), 20% methanol (5 mL), and 100% methanol (5 mL). Then, the eluting solution of 100% methanol was evaporated to dryness under reduced pressure and dissolved with methanol to a volume of 2 mL. Finally, the methanol solution was further filtered through a 0.45 µm nylon membrane for HPLC analysis.

### 3.4. Method Validation

#### 3.4.1. Linearity, LOD, and LOQ

Mixed standards of a series of appropriate concentration gradients were prepared, and the peak areas of each constituent were analyzed under our optimum chromatographic condition. The LODs and LOQs are the lowest concentrations of target analytes that can be detected and quantified at a signal-to-noise ratio of 3 and 10, respectively [50].

#### 3.4.2. Precision

The precision was determined by mixed standard solutions of three different concentration levels within the range of calibration curves. The intra-day precision was obtained by analyzing these samples four times in 1 d. The inter-day precision was obtained by analyzing these samples once a day for three consecutive days. The RSD of the peak area was taken as a measure of precision.

#### 3.4.3. Repeatability and Stability

The repeatability was confirmed with the tuber root powder of ZOJ in nine parallel experiments under the optimum IL-UAE preparation and chromatographic conditions. Additionally, the sample stability was also determined at different times over three consecutive days. The RSDs of the peak area were calculated as the index of repeatability and stability.

#### 3.4.4. Accuracy

To evaluate the accuracy, a pre-analyzed ZOJ tuber root sample was spiked with mixed standard at three different concentration levels (50, 100, and 150%) of this known sample. The mixtures were then analyzed by the proposed sample preparation and chromatographic method. Each concentration level was performed in triplicate. Accuracy was calculated as the recovery (%) of the target standard.

## 4. Conclusions

In this study, an efficient, green, and reliable method was developed for the simultaneous extraction and determination of four characteristic steroidal saponins and homoisoflavonoids by IL-UAE combined with HPLC-DAD-ELSD analysis. This work will be helpful for the quality control of ZOJ. It can also present a promising reference for the simultaneous extraction and determination of different kinds of compounds in other medicinal plants.

## Figures and Tables

**Table 1:** Regression equation, correlation coefficient, linear ranges, LOQ, and LOD of four analytes.

Analyte	Regression Equation	R^2^	Linear Range (μg/mL)	LOD (μg/mL)	LOQ (μg/mL)
S1	logY = 1.2603 logX + 3.861	0.9992	15.00 – 75.00	1.80	4.80
S2	logY = 1.2236 logX + 3.831	0.9978	6.76 – 33.80	2.16	5.41
H1	Y = 42276X + 10292	0.9999	6.90 – 34.50	0.07	0.28
H2	Y = 38451X + 28209	0.9999	17.16 – 85.80	0.10	0.34

**Table 2:** Inter- and intra-day precision, repeatability, and stability of four analytes (RSD, %).

Analyte	Precision			Repeatability (*n* = 9)	Stability (*n* = 9, 3 Days)
	Level	Inter-Day (*n* = 4)	Intra-Day (*n* = 9)		
S1	Low	3.57	3.35	3.84	3.73
	Medium	2.54	1.83		
	High	2.69	4.71		
S2	Low	4.63	4.05	4.97	4.62
	Medium	4.46	1.28		
	High	3.75	3.05		
H1	Low	4.38	1.27	3.07	3.72
	Medium	2.57	2.21		
	High	2.13	2.62		
H2	Low	4.45	1.29	1.82	2.85
	Medium	2.62	3.48		
	High	2.13	2.60		

**Table 3:** Analytical results of real samples of ZOJ (mg/g).

Samples	Parts	Origin *	S1	S2	H1	H2
1	Tuber roots	Chongshou	0.1742 ± 0.0085	0.0709 ± 0.0083	0.1350 ± 0.0030	0.3251 ± 0.0051
2		Kandun	0.2174 ± 0.0058	0.0857 ± 0.0101	0.1409 ± 0.0057	0.3407 ± 0.0099
3		Shengshan	0.2250 ± 0.0100	0.1015 ± 0.0107	0.1442 ± 0.0029	0.3242 ± 0.0037
4	Fibrous roots	Chongshou	0.4693 ± 0.0184	0.3518 ± 0.0211	0.3099 ± 0.0086	0.5278 ± 0.0145
5		Kandun	0.4941 ± 0.0026	0.2933 ± 0.0027	0.2602 ± 0.0077	0.4141 ± 0.0089
6		Shengshan	0.5313 ± 0.0298	0.3354 ± 0.0278	0.2784 ± 0.0059	0.4919 ± 0.0088

* Towns in Cixi City, Zhejiang province, China.

## Data Availability

The data presented in this paper are available on request from the corresponding authors.

## Supplementary Materials

The following supporting information can be downloaded at: https://www.mdpi.com/article/10.3390/molecules27217380/s1, Table S1: Recoveries of four analytes.
