# Supplementary material for: Simultaneous Extraction and Determination of Characteristic Steroidal Saponins and Homoisoflavonoids in Zhejiang Ophiopogon japonicus
Source: Molecules. 2022 Oct 30;27(21):7380. doi: 10.3390/molecules27217380 (PMC9656867; doi:10.3390/molecules27217380)
Supplement: Supplementary file 1 [file molecules-27-07380-s001.zip › molecules-1985119-supplementary.pdf]

Supporting Information

Simultaneous extraction and determination of characteristic steroidal saponins and homoisoflavonoids in Zhejiang *Ophiopogon japonicus*

Yaoyao Zhu[1], Liling Wang[2], Meixu Chen[1], Yifeng Zhou[1,3,4] * and Jun Huang[1,3,4] *

[1] School of Biological and Chemical Engineering, Zhejiang University of Science and Technology, Hangzhou 310023, China

[2] Zhejiang Academy of Forestry, Hangzhou 310023, China

[3]Zhejiang Provincial Key Lab for Chemical and Biological Processing Technology of Farm Produces, Hangzhou, 310023, China

[4]Zhejiang Province Collaborative Innovation Center of Agricultural Biological Resources Biochemical Manufacuring, Hangzhou, 310023, China

* Correspondence: yfzhou@zust.edu.cn(Y.Z.); huangjun@zust.edu.cn(J.H.)

Table S1. Recoveries of four analytes.

Analyte	Original (mg/g)	Added (mg/g)	Detected (mg/g)	Recovery (%)	Average recovery rate (%)	RSD (%)
			0.1937	80.02		
		0.0672	0.1991	88.04	83.88	1.38
			0.1961	83.59		
			0.2607	89.88		
**S1**	0.1399	0.1344	0.2743	100.00	96.91	3.03
			0.2754	100.83		
			0.3243	91.47		
		0.2016	0.3177	88.19	90.50	1.26
			0.3251	91.85		
			0.1217	114.83		
		0.0397	0.1151	98.33	103.80	3.23
			0.1151	98.23		
			0.1528	96.60		
**S2**	0.0761	0.0794	0.1421	83.18	88.08	4.03
			0.1432	84.45		
			0.1852	91.71		
		0.1190	0.1752	83.26	86.54	3.01
			0.1768	84.65		
			0.1460	117.11		
		0.0460	0.1397	103.57	106.76	2.99
			0.1379	99.60		
			0.1818	97.44		
**H1**	0.0921	0.0921	0.1781	93.41	97.44	2.04
			0.1855	101.47		
			0.2281	98.50		
		0.1381	0.2373	105.15	102.93	2.26
			0.2373	105.16		
			0.4663	89.82		
		0.1564	0.4798	98.48	95.84	1.72
			0.4810	99.21		
			0.5778	88.47		
**H2**	0.3258	0.2848	0.6193	103.06	95.34	3.50
			0.5949	94.48		
			0.7506	99.45		
		0.4272	0.7892	108.47	100.28	4.42
			0.7228	92.92		
